# Natural diversity of cellulases, xylanases, and chitinases in bacteria

**DOI:** 10.1186/s13068-016-0538-6

**Published:** 2016-06-29

**Authors:** Darrian Talamantes, Nazmehr Biabini, Hoang Dang, Kenza Abdoun, Renaud Berlemont

**Affiliations:** Department of Biological Sciences, California State University, Long Beach, 1250 Bellflower Blvd., Long Beach, 90840-9502 USA

**Keywords:** CAZy, Carbohydrate, Glycoside hydrolase, Polysaccharide, Biofuel, Cellulase, Xylanase, Chitinases, GH

## Abstract

**Background:**

Glycoside hydrolases (GH) targeting cellulose, xylan, and chitin are common in the bacterial genomes that have been sequenced. Little is known, however, about the architecture of multi-domain and multi-activity glycoside hydrolases. In these enzymes, combined catalytic domains act synergistically and thus display overall improved catalytic efficiency, making these proteins of high interest for the biofuel technology industry.

**Results:**

Here, we identify the domain organization in 40,946 proteins targeting cellulose, xylan, and chitin derived from 11,953 sequenced bacterial genomes. These bacteria are known to be capable, or to have the potential, to degrade polysaccharides, or are newly identified potential degraders (e.g., *Actinospica*, *Hamadaea*, *Cystobacter*, and *Microbispora*). Most of the proteins we identified contain a single catalytic domain that is frequently associated with an accessory non-catalytic domain. Regarding multi-domain proteins, we found that many bacterial strains have unique GH protein architectures and that the overall protein organization is not conserved across most genera. We identified 217 multi-activity proteins with at least two GH domains for cellulose, xylan, and chitin. Of these proteins, 211 have GH domains targeting similar or associated substrates (i.e., cellulose and xylan), whereas only six proteins target both cellulose and chitin. Fifty-two percent of multi-activity GHs are hetero-GHs. Finally, GH6, −10, −44 and −48 domains were mostly C-terminal; GH9, −11, −12, and −18 were mostly N-terminal; and GH5 domains were either N- or C-terminal.

**Conclusion:**

We identified 40,946 multi-domain/multi-activity proteins targeting cellulase, chitinase, and xylanase in bacterial genomes and proposed new candidate lineages and protein architectures for carbohydrate processing that may play a role in biofuel production.

**Electronic supplementary material:**

The online version of this article (doi:10.1186/s13068-016-0538-6) contains supplementary material, which is available to authorized users.

## Background

Glycoside hydrolases (GH) are key enzymes for the processing of complex carbohydrates [1]. Plant-derived cellulose and xylan represent the major source of carbon in terrestrial ecosystems, whereas chitin is the most abundant source of carbon in marine ecosystems. The deconstruction of these polysaccharides by GH is key to the global earth carbon cycle [2], mammal nutrition [3], and is the primary target of several industries (e.g., biofuel production) [4]. Many GH families for carbohydrate processing have been identified, some being populated with many identified and characterized proteins (e.g., cellulases from GH5) and others containing few sequences (e.g., arabinases from GH93) [1]. Sixty-one GH families have been assigned a PFam ID, allowing for domain identification based on HMM-profile recognition [5, 6]. According to characterized proteins in the CAZy database (http://www.cazy.org), many GH families display substrate specificity, and so the potential activity of the GH can be determined by examining the protein sequence. For example, most enzymes from GH families 5, 6, 7, 8, 12, 44, 45, and 48 act on cellulose, while GH families 10, 11, and 30 are mostly xylanases, and GH family 18, 19, and 85 are chitinases [1, 7]. There are also some GH families that do not target-specific substrates (e.g., GH16).

The complete breakdown of polysaccharide requires the synergistic action of multiple enzymes acting on internal bonds (e.g., endocellulase), extremities (e.g., exocellulase), and intermediate degradation products (e.g., β-glucosidase). Thus, most identified degrader lineages have several genes coding for GH and many seemingly redundant enzymes targeting similar substrates [1, 7, 8]. Across environments, polysaccharides are associated and form complex structures (e.g., plant cell walls); therefore, many degraders often target several substrates (e.g., cellulose and xylan) [7, 9]. To degrade complex polysaccharides, bacteria have adopted several strategies including the production of (i) individual enzymes, sometimes associated with non-catalytic accessory domains, such as carbohydrate-binding modules (CBM) [8]; (ii) production of complex proteins with multiple GH domains (i.e., multi-activity GH, MAGH), with or without CBM [10]; and (iii) the production of non-covalent multi-protein complexes called cellulosomes [4]. When released simultaneously, distinct GH domains act synergistically and display overall improved hydrolytic activity, compared to single domains. Synergy among GH domains is further achieved by the physical association of catalytic domains into complex proteins with multiple catalytic domains, and in cellulosomes [4, 10]. These MAGHs and protein complexes are promising tools for improving biomass processing [4, 10–12].

A particular bacteria’s potential ability to deconstruct polysaccharides can be predicted by the number and diversity of GH domains in its genome [7, 13]. In sequenced bacterial genomes, the presence of GHs is mostly conserved at the genus level [7, 9]; therefore, the presence of GH domains in new members of previously identified genera can be easily inferred. Little is known, however, about the conservatism of GH domain organization across bacteria. To address this question, we developed a custom bioinformatic pipeline aimed at identifying and listing the protein architectures (aka the domain organization) for GHs targeting cellulose, xylan, and chitin in sequenced bacterial genomes. Next, we analyzed the conservatism of domain organization in MAGHs, and investigated the variability of domain organization in identified polysaccharide degraders (i.e., bacterial lineages associated with GH for polysaccharide degradation). We hypothesized that, across bacterial gnomes, the distribution of GH domains and the architecture of proteins with GH domains would correlate. Indeed, several groups of bacteria are systematically identified as polysaccharide degraders [8, 14, 15] and the distribution of GH domains in sequenced bacterial genomes is phylogeneticaly conserved at the genus level [7, 9]. Thus, one could expect that bacteria from the same genus, with similar GH content, share similar GH organization. Finally, we specifically investigated the association of GH domains in MAGHs. We expected a high frequency of MAGHs with synergistic domains, (i.e., targeting the same substrate) and/or domains targeting physically associated substrates (i.e., cellulose and xylan in plant cell walls). MAGHs with a combination of catalytic domains that target the same substrate and/or physically associated substrates would benefit from identical regulation and expression processes and increase the synergy between catalytic domains by reducing their diffusion [16], among other benefits. Conversely, we expected that there would be few proteins with GH domains targeting unrelated substrates (e.g., cellulose:chitin or xylan:chitin).

Our systematic investigation of the association and organization of catalytic and accessory domains involved in carbohydrate processing across sequenced bacterial genomes highlights new proteins, new domain architectures, and provide new insights about how bacteria are able to process complex carbohydrates with implications for biofuel research.

## Results

### Distribution of GH for cellulose, xylan, and chitin

We searched 11,953 sequenced genomes and identified 40,946 proteins containing 41,196 domains that target cellulose, xylan, or chitin (Additional file 1). First, 25,682 identified proteins were single domain (Table 1) with no accessory domain. Next, 15,047 proteins were multi-domain proteins with a unique GH domain (i.e., MDGH) targeting cellulose, xylan, or chitin, associated with other domains (e.g., CBM). Finally, 217 multi-activity proteins (i.e., MAGH) had multiple catalytic domains for cellulose, xylan, or chitin, along with accessory domains.

**Table 1:** Distribution of identified GH domains and multi-activity GHs (i.e., MA-GHs) in sequenced bacterial genomes

GH	PFAM	Sub.	#DOM.	#Prot.	CAZy	MA-GHs (2nd GH domain)											Figure
						5	6	8	9	10	11	12	18	19	44	48	
5	PF00150	Cel.	7908	7885	4907	*23*	3		2	2	7	41	5		3		Additional file 2: Figure S1
6	PF01341	Cel.	3088	3087	534	3	*1*		3			1					Additional file 3: Figure S2
8	PF01270	Cel.	5003	5003	1738								1				Additional file 4: Figure S3
9	PF00759	Cel.	2307	2306	878	2	3		*1*						8	12	Additional file 5: Figure S4
10	PF00331	Xyl.	2541	2535	1579	2				*6*	20					2	Additional file 6: Figure S5
11	PF00457	Xyl.	534	507	586	7				20	*22*						Additional file 7: Figure S6
12	PF01670	Cel.	2515	2515	368	41	1										Additional file 8: Figure S7
18	PF00704	Chi.	12,771	12,715	5929	5		1					*51*	2			Additional file 9: Figure S8
19	PF00182	Chi.	1683	1683	1989								2				Additional file 10: Figure S9
30	PF02055	Xyl.	1361	1361	933												
44	PF12891	Cel.	143	143	104	3			8								Additional file 11: Figure S10
45	PF02015	Cel.	31	31	18												
48	PF02011	Cel.	346	345	724				12	2						*1*	Additional file 12: Figure S11
85	PF03644	Chi.	941	941	246												

*Sub.* substrate targeted by the GH domain, *Cel.* cellulose, *Xyl* xylan, and *Chi.* chitin. *#DOM.* number of identified domain and *#Prot.* number of proteins identified in this study, *CAZy* number of identified domain in bacteria according to the CAZy database (http://www.cazy.org, as of March 2016)

Homo-GHs are in italics

**Fig. 1:**
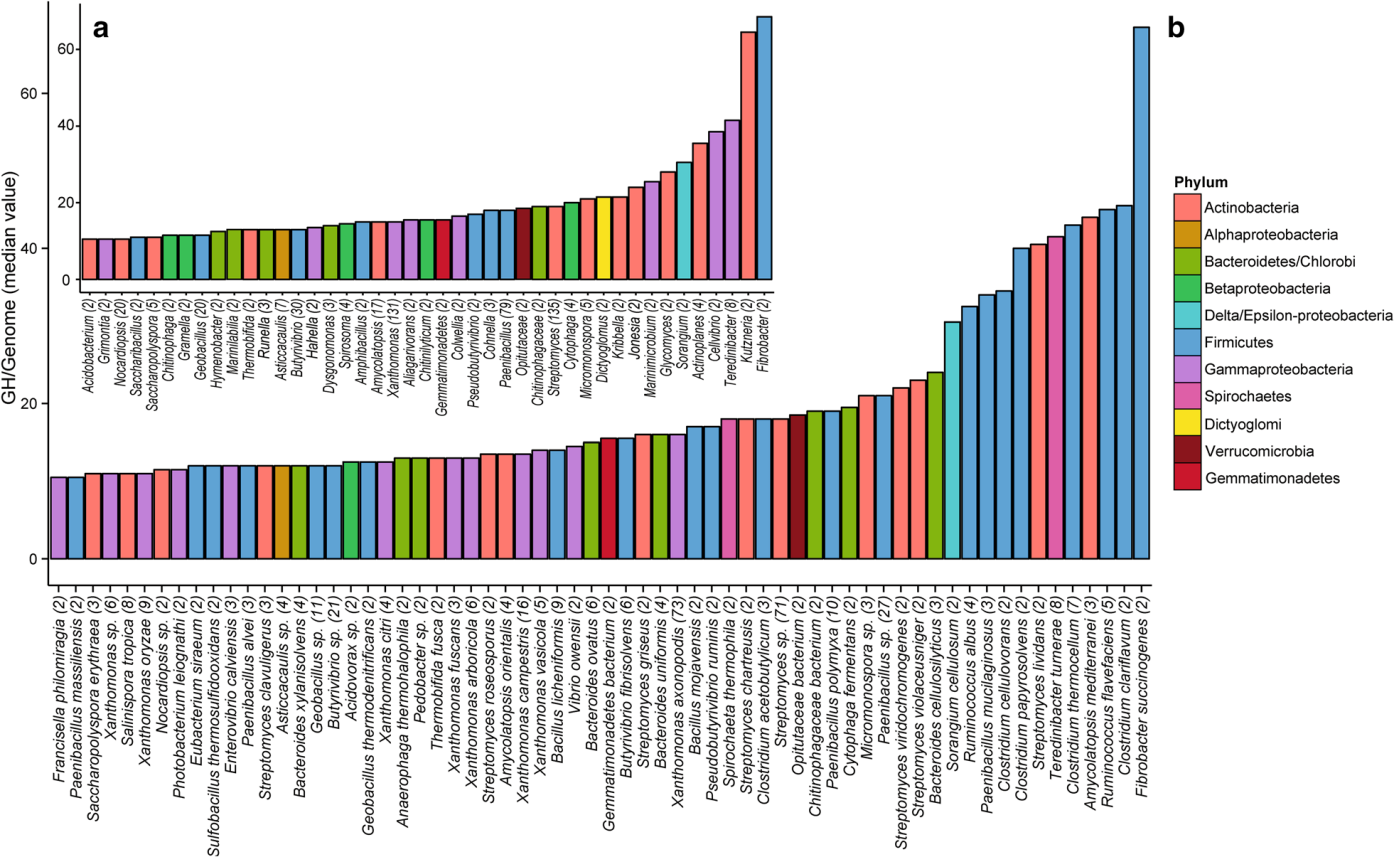
Frequency of GHs targeting cellulose, xylan, and chitin per sequenced genome (number of analyzed genomes in parentheses), **a** in bacterial genera and **b** species

To identify bacteria with a high potential for cellulose, xylan, and chitin processing, we first investigated the average frequency of GH domains for cellulose, xylan, and chitin per genome, at the genus and species levels (Fig. 1).

**Fig. 2:**
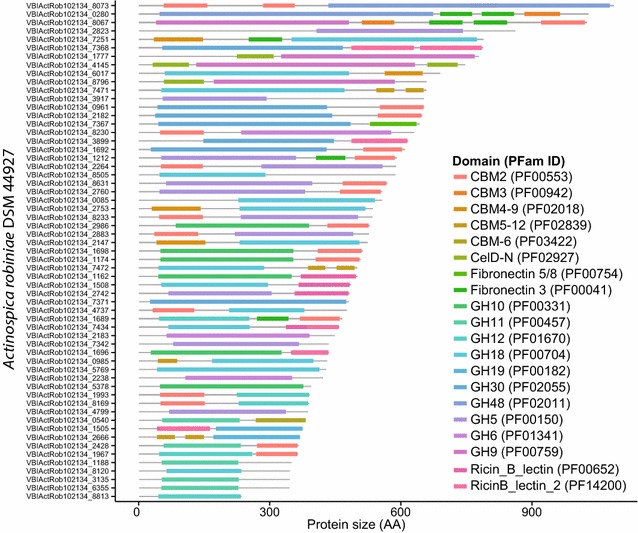
Architecture of 59 proteins with GH domains targeting cellulose, xylan, and chitin in *Actinospica robiniae* DSM44927 (phylum Actinobacteria). There are no genomic sequences of close relatives to *A*. *robiniae*

Some well-known degrader genera consistently contained > 8 GHs of interest per genome (e.g., *Fibrobacter, Streptomyces, Xanthomonas*). In contrast, some genera, although associated with known degraders, contained few GHs per genomes on average (e.g., *Clostridium, Ruminococcus*) (Fig. 1a). We also identified several lineages that had not been previously known to be cellulose, xylan, and chitin degraders, but contained GHs that target these carbohydrates. Among others, *Opitutaceae* (phylum Verrucomicrobia, Additional file 13: Figure S12), *Amycolatopsis* and *Micromonospora* (phylum Actinobacteria, Additional file 14: Figure S13, Additional file 15: Figure S14), *Spirochaeta* (phylum Spirochaeta, Additional file 16: Figure S15), *Anaerophaga* (phylum Bacteroides, Additional file 17: Figure S16), and *Asticcacaulis* (phylum Alphaproteobacteria, Additional file 18: Figure S17) displayed many sequences for GHs targeting cellulose, xylan, and chitin. For example, *Opitutaceae* (*n* = 2 sequenced genomes) contained 19 or 18 proteins with GH targeting cellulose, xylan and chitin. In these 2 genomes, most proteins were single-domain proteins targeting cellulose (i.e., many GH5 and few GH8), xylan (i.e., GH10), and, in *O. bacterium* TAV5, two chitinases from GH18 (Additional file 13: Figure S12). *Amycolatopsismediterranei* U32 contained many multi-domain proteins, including some MA-GHs and GH domains associated with lipases (Additional file 14: Figure S13). This suggests that, at the genus level, some taxa have higher potential for cellulose, xylan, and chitin processing (e.g., *Clostridium, Ruminococcus*) than previously thought, and that many poorly characterized genera deserve further attention. However, several species from various genera were associated with >8 GHs of interest per genome (e.g., *Clostridium clariflavum, C. thermocellum, Ruminoccocus flavefaciens, R. albus*) (Fig. 1b). Finally, we identified a set of phylogeneticaly isolated species, with no close sequenced relatives, but with a high potential for cellulose, xylan, and chitin processing. For example, species from the genera *Actinospica* (Fig. 2), *Hamadaea* (Additional file 19: Figure S18), *Cystobacter* (Additional file 20: Figure S19), *Catelliglobosispora* (Additional file 21: Figure S20), *Sporocytophaga* (Additional file 22: Figure S21), *Kitasatospora* (Additional file 23: Figure S22), *Niastella* (Additional file 24: Figure S23), and *Microbispora* (Additional file 25: Figure S24), among others, have no close sequenced relative and a high potential for cellulose, xylan, and chitin deconstruction (i.e., >20GHs for cellulose, xylan, and chitin per genome).

### Conservatism of protein architecture

Excluding unique domains, observed once, we identified 210 types of protein domains associated with the GH domains of interest (Additional file 26: Table S1). First, we identified 18 additional types of GH domains targeting oligosaccharides (e.g., GH2, 3) and other sugars (e.g., mannanase from GH26, galactosidase from GH35). Next, we identified other catalytic domains, including glycosyltransferase (mostly GT2), polysaccharide deacetylase, some lipases and esterases (e.g., GDSL), and few alpha/beta-hydrolases. We also identified many non-catalytic domains, including 13,573 CBMs from 17 families and targeting cellulose (e.g., CBM2, 3), xylan (e.g., CBM35), and chitin (e.g., CBM5_12). Next, we identified 1102 dockerins (i.e., PF00404) and 7 cohesins (i.e., PF00963) for cellulosome associated with GHs. Finally, 478 domains of unknown function (DUF), 60 bacterial neuraminidase domains (i.e., BNR, PF02012, PF14873), 727 S-layer homology domains (i.e., SLH, PF00395), 2597 fibronectin domains (i.e., PF00041, PF16893), 1308 lectin domains (e.g., PF14873, PF11721), and 23 Cadherin-like domains (i.e., PF12733, PF00028, PF16184) were identified, among others. With the exception of CBMs and some lectins, most of these domains were not listed in the CAZy database. However, their high frequency in association with GHs for cellulose, xylan, and chitin suggested that these accessory domains could have functional or structural implications in carbohydrate processing.

**Fig. 3:**
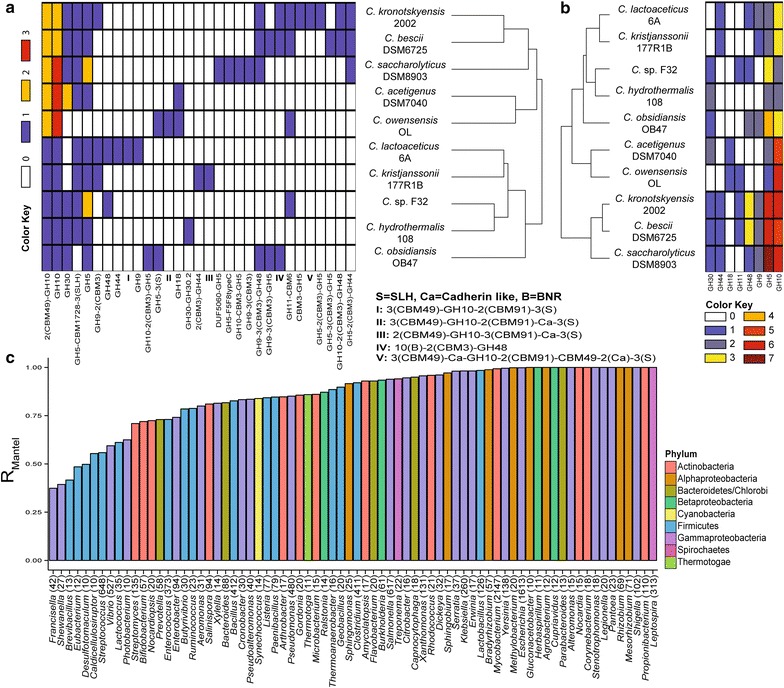
Example of clustering of 10 strains from the genus *Caldicellulosiruptor*, according to (**a**) the distribution of proteins with GH domains for cellulose, xylan, chitin, and accessory non-catalytic domains, and (**b**) the distribution of GH domains for cellulose, xylan, and chitin. *Color key*: number of identified protein (**a**) and GH domain (**b**) in each analyzed genome. **c** Significant correlations between the A and B clustering analyses for bacterial genera with at least 10 sequenced genomes

We next tested the conservatism of protein architecture in genera with >3 sequenced genomes by clustering the genomes based on the architecture of proteins with GH domains (accounting for all the accessory domains) and, in a separate analysis, the distribution of GH domains only (Fig. 3; Additional file 27: Table S2). First, in few genera including *Cellulomonas* (*n* = 5 genomes) and *Cytophaga* (*n* = 4 genomes), the clustering based on protein architecture did not correlate with the distribution of GH domains. Next, in some genera, including *Caldicellulosiruptor* (*n* = 10 genomes), the clustering based on protein architecture correlated partially with the distribution of GH domains (*P*_mantel_ = 0.002, *r*_mantel_ = 0.55)(Fig. 3a, b). Finally, in many genera the two clusterings were highly consistent (Fig. 3c; Additional file 17: Table S2). For example, in *Xanthomonas* (*n* = 131 genomes), the clustering of strains based on proteins targeting cellulose, xylan, chitin, and their accessory domains correlated with the clustering based on the distribution of GH domains (*P*_mantel_ = 0.001, *r*_mantel_ = 0.96, Fig. 3c; Additional file 27: Table S2; Additional file 28: Figure S25). Significant correlations were independent of the number of sequenced genomes and unaffected by the number of GHs per genome.

Across sequenced bacterial genomes, many proteins contained accessory non-catalytic domains. Within genera, bacteria shared sets of simple proteins with similar architectures. However, the more complex protein architectures were species specific (e.g., GH10-CBM3-GH5 in *Caldicellulosiruptor saccharolyticus* DSM8903)(Fig. 3a). The distribution of these unique proteins had no effect on the architecture-based clustering. Finally, complex protein architectures were conserved in few genera [e.g., 41 (GH5)-(Ricin-B-lectin)-(GH12) in the genus *Sallinispora*] (Additional file 2-12: Figures S1, Additional file 3: Figure S2, Additional file 4: Figure S3, Additional file 5: Figure S4, Additional file 6: Figure S5, Additional file 7: Figure S6, Additional file 8: Figure S7, Additional file 9: Figure S8, Additional file 10: Figure S9, Additional file 11: Figure S10, Additional file 12: Figure S11).

### Architecture of multi-activity GH

**Fig. 4:**
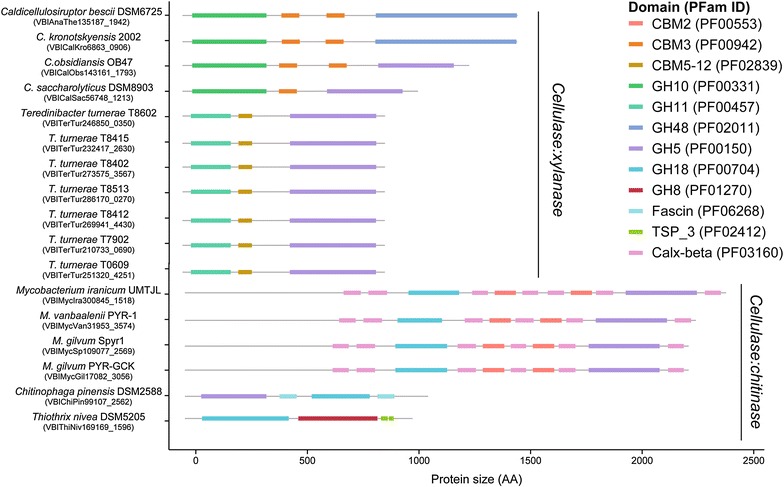
Architecture of multi-activity GHs (i.e., MA-GHs) targeting cellulose:xylan and cellulose:chitin

Among identified proteins with at least one GH domain of interest, 40,729 had a single GH domain or a single GH domain associated with non-catalytic accessory domain(s). However, we identified 217 proteins with multiple GH domains that targeted some combination of cellulose, xylan, or chitin (i.e., MA-GH). One hundred and five MA-GHs had 2 GH domains from the same family (e.g., GH5-GH5), whereas 112 MA-GHs had different GH domains (Table 1). More precisely, 99 MA-GHs were cellulase:cellulase (i.e., 26 homo-GH–same GH domain- vs. 73 hetero-GH–distinct GH domains), 53 MA-GHs were chitinase:chitinase (i.e., 51 homo-GH vs. 2 hetero-GH), and 48 MA-GHs were xylanase:xylanase (i.e., 28 homo-GH vs. 20 hetero-GH). We identified 11 MA-GHs as cellulase:xylanase in *Caldicellulosiruptor* and *Teredinibacter* and 6 MA-GHs were cellulase:chitinases from *Mycobacteria*, *Chitinophaga*, and *Thiotrix* (Fig. 4; Table 1). We identified 16 proteins with 3 GH domains targeting xylan and chitin in *Rumniococcus* and *Paenibacillus*, among others (Additional file 29: Figure S26). 10 of these proteins were homo-GH whereas 5 xylanases and 1 chitinase were hetero-GH. In addition, some long xylanases displayed an extra catalytic domain for polysaccharide deacetylase (i.e., PF01522).

MA-GHs contained catalytic domains from GH families 5, 6, 9, 10, 11, 12, 18, and 44. In contrast, GH domains from families 8, 19, 30, 45, and 85 were rarely observed in association with other GH domains. GH domains from families 5, 9, 12, 44, and 48 were found in hetero-GHs, whereas GH 11 and 18 formed mostly homo-GHs.

In MA-GHs, domains from families 6, 10, 44 and 48 were predominantly located in the C-terminal end of the protein, whereas domains from GH9, 11, 12, and 18 were mostly located at the N-terminal end of MA-GH. Finally, GH5 domains were located close to the N- or C-terminal ends (Additional file 2: Figures S1, Additional file 3: Figure S2, Additional file 4: Figure S3, Additional file 5: Figure S4, Additional file 6: Figure S5, Additional file 7: Figure S6, Additional file 8: Figure S7, Additional file 9: Figure S8, Additional file 10: Figure S9, Additional file 11: Figure S10, Additional file 12: Figure S11, Additional file 29: Figure S26).

## Discussion

Protein domains are defined as “conserved, functionally independent protein sequences that bind or process ligands using a core structural motif” [17]. Although many proteins are known to be multi-domain assemblages [18], most studies of proteins are focused on individual domains and do not consider how interactions between domains might affect the structure and the activity of enzymes. The selection pressure for the domain combination is governed by the structural (see [19] for review) and the functional advantage provided to the organism. Indeed, multi-domain proteins connect complementary domains and activities. Thus, analyzing the architecture of GHs that target cellulose, xylan, and chitin in bacterial genomes allows us to further understand the distribution of GH domains [7, 9], highlight the association of GHs with CBMs (i.e., carbohydrate-binding modules) and other catalytic and non-catalytic accessory domains, and provides an understanding of how bacteria degrade carbohydrates.

Our HMM-based survey of bacterial genomes reveals the variability and the distribution of GH architecture in well-known degrader genera (e.g., *Clostridium*, *Ruminococcus*) and avoids biased interpretation of bacterial carbohydrate processing based on known, or predicted, hydrolytic capabilities. To the best of our knowledge, the identified cellulases, xylanases, and chitinases described here outnumbered currently available databases (Table 1) and uncover the high potential for carbohydrate processing in lineages not included in previous studies (e.g., *Actinospica*) [1, 13]. However, there are a number of caveats associated with our approach to studying the diversity of enzymes involved in carbohydrate processing across bacteria. We recognize that some GH genes we identified as potential cellulases, xylanases, and chitinases may have other enzymatic functions given that some GHs have side activities (e.g., [20]). In addition, some enzymes identified as cellulases are instead involved in cellulose biosynthesis or in the interaction between microorganisms and plants (e.g., GH8) [9, 13, 21].

Among the identified accessory domains, CBMs anchor GH domains onto carbohydrates (i.e., targeting effect), increase the local concentration of catalytic domains (i.e., proximity effect) [22], and sometimes disrupt the substrate (i.e., disruptive effect, e.g., [23]). The distribution of CBMs in association with GHs for cellulose, xylan, and chitin mirrors the distribution of GHs in sequenced bacterial genomes. Here, we listed 4072 CBMs targeting cellulose (e.g., CBM2, 3, and 6), 5967 CBMs targeting chitin (e.g., CBM5_12), 448 CBMs for xylan (e.g., CBM9, CBM35), and 1293 CBM4_9 targeting mostly xylan and sometimes cellulose. In addition, few CBMs for starch (i.e., CBM20) and mannose (i.e., CBM27) were also associated with GHs for cellulose, xylan, and chitin. Besides CBMs, other identified accessory domains include, among others: (i) Lectins, known as carbohydrate-recognition proteins and frequently associated with glycosidases or proteases and included in the CBM classification (e.g., CBM13) [24, 25]; (ii) SLHs (i.e., S-layer homology domains) interacting with bacterial cell wall carbohydrates (e.g., peptidoglycan) [26, 27] and thus potentially anchoring associated catalytic domain onto the cell wall and possibly interacting with the substrate of GHs [27]; (iii) Bacterial neuraminidases involved in the hydrolysis of glycoconjugates (e.g., α-2,3-linked sialic acids) and associated with biofilm production [28]; (iv) Cadherin domains involved in cell–cell adhesion and known as potential Ca-dependent carbohydrate-binding modules (i.e., Ca-CBM) [29]. Globally, many of these accessory domains, although not listed as CBMs [1], display affinity for carbohydrates, are likely to affect the carbohydrate processing by GHs [27, 30] and could be listed as CBMs. The distribution of these accessory domains is not random as some genomes display mostly complex multi-domain GHs [e.g., *A. robineae* DSM 44927 (Fig. 2), *A. mediterranei* U32 (Additional file 14: Figure S13), and *H. tsunoensis* DSM44101 (Additional file 19: Figure S18)] or both single and multi-domain GHs [e.g., *C. fuscus* DSM2262 (Additional file 20: Figure S19)], whereas other lineages contain almost exclusively single-domain GHs [e.g., *O. bacterium* TAV1 (Additional file 13: Figure S12)]. Complex multi-domain GHs, with CBMs, outperform single-domain GHs in diluted environments but not in concentrated systems [31]. This suggests that the systematic association of GHs with CBMs, broadly defined, could reflect the adaption of bacteria to specific environments (e.g., marine vs. soil ecosystems).

Our analysis of the distribution of GH architectures across sequenced genomes supports the hypothesis that the distribution of GH domains and the potential for carbohydrate processing are taxonomically conserved [7, 9]. Many genera (e.g., *Stenotrophomas*, *Rhizobium*, *Gluconoacetobacter*) displayed conserved protein architectures. In these genomes, knowing the exact protein architectures provides a way to estimate the GH content and the protein architecture in newly identified strains. However, beyond conserved sets of single-domain proteins, many bacteria display species-specific protein architectures. These unique protein architectures have no effect on the clustering of genomes and their domain organization cannot be predicted. This highlights the multimodularity of GH and suggests the rapid evolution of closely related organisms regarding their potential to target substrates in the environment. Thus, using our data, it is possible to infer the GH content of taxonomically identified bacteria and complex microbial communities (e.g., metagenomes). Because of the extensive variation even between closely related strains, however, inferring the exact protein architecture will remain a major challenge.

Multi-activity proteins (i.e., MAGHs) mainly correspond to associations of GH domains targeting similar substrates (e.g., cellulase:cellulase). In addition, most MAGHs are homo-GHs (e.g., GH5-GH5). The association of two identical GH domains into MAGHs suggests a duplication-fusion of the catalytic domain, whereas the rare hetero-GHs (e.g., GH5-GH6) result from more complex recombination [32, 33]. Thus, bacteria target one substrate at a time and take advantage of the synergistic activity among catalytic domain targeting similar substrate [8]. This allows for precise regulation of each pathway for carbohydrate deconstruction as observed in few bacterial lineages (e.g., *Streptomyces* [34] and filamentous fungi [35]).

The mode of action of the GHs combined in MAGHs is key to elucidating the synergy among catalytic domains [36]. Most MAGHs have GHs with identical, expected, modes of action (e.g., endocellulase-endocelluase). However, combining endo-type and exo-type of GH (e.g., GH9-3(CBM3)-GH48 in *C. bescii*) produced unexpected enzymatic activity [10]. In this context, listing the architectures of MAGHs will help identify interesting proteins [e.g., GH6-CBM3-fn3-GH12-CBM2, GuxA, Additional file 3: Figure S2  (see US Patent US 20030104522 A1)] and candidate proteins to be tested [e.g., GH9-3(CBM3)-GH5 and GH10-CBM3-GH5, both from *Caldicellulosiruptor*, (Fig. 4; Additional file 2: Figure S1) and CBM4/9-CelD-GH9-GH6 from *Kribella* (Additional file 3: ​Figure S2)].

## Conclusions

In the environment, microbes (i.e., fungi and bacteria) are essential for the deconstruction of complex carbohydrates (e.g., cellulose) [37]. The increasing number of sequenced genomes, mostly from bacteria, and their consistent annotation [38], provides an unprecedented opportunity to perform large-scale comparative genomics [9, 39, 40]. Our systematic investigation of sequenced bacterial genomes to identify protein architectures has many potential uses. First, it provides an overview of the spatial organization of catalytic domains (i.e., GHs) and their association with CBMs, as well as other non-catalytic accessory domains involved in carbohydrate binding. Second, our analysis reveals the heterogeneous distribution of GHs in bacteria. Indeed, although GH domains are conserved within bacterial genera [7, 9], the complex domain architectures are mostly species specific. Thus, knowing the phylogenetic distribution and the association between catalytic domains targeting the major carbohydrates, it will be possible to predict the GH content in most bacteria. This will help identify new bacterial isolates with increased potential for carbohydrate processing. However, the GH architecture remains extremely variable and thus cannot be predicted. Finally, listing the GH architectures will serve as a guide for future tests on the taxonomic breadth of domains association and their spatial organization.

## Methods

### GH identification

Protein sequences from sequenced bacterial genomes were retrieved from the PATRIC database [41] and analyzed using a custom bioinformatic pipeline aimed at identifying proteins involved in cellulose, xylan, and chitin processing. Briefly, bacterial proteins with GH domains targeting cellulose, xylan, and chitin were identified using a custom database of hidden Markov Model profiles, retrieved from PFam-A [6]. Then, selected proteins with GHs for cellulose, xylan, or chitin were analyzed against the entire PFAM-A database (as of December, 2015) to confirm the GH domains and identify their associated domains (e.g., CBMs). Identified domains with e value <10^−5^ and alignment coverage >60 % of PFam length were used in subsequent analyses. Substrate specificity of identified GH and CBM domains was derived from biochemically characterized bacterial homologs found in the CAZy database [1, 7]: GH 5, 6, 7, 8, 9, 12, 44, 45, and 48 were identified as cellulase; GH 10, 11, and 30 were identified as xylanase; and GH 18, 19, and 85 were identified as chitinases. Some GH families identified recently (e.g., GH74), have no assigned HMM and thus are not included in this study. Sequences of interest can be retrieved directly from the database using the listed IDs (e.g., VBIactrob102134_8073) in figures and supplementary data and the PATRIC portal (https://www.patricbrc.org/portal/portal/patric/Home) [41]. Finally, the complete taxonomy of each individual strain was retrieved from the NCBI taxonomy server (http://www.ncbi.nlm.nih.gov/Taxonomy/).

### Statistical analysis

GH distribution and domain organization in sequenced bacterial genomes were analyzed using Vegan, Stats, and APE packages in the R software environment [42, 43]. Clustering bacterial strains used two distinct approaches. First, genomes were clustered according to the distribution of GH domains per genome, regardless of the protein architecture. Second, we compared the architecture of all identified proteins with GH domains for cellulose, xylan and chitin, including accessory domains, and then clustered the sequenced genomes as described before. To investigate correlation among clusterings based on the number of sequenced genomes in a particular bacterial lineage or the number of GH domains within a genome, we performed Mantel correlation tests (999 permutations) on distance matrixes used for clustering.

## Additional files

10.1186/s13068-016-0538-6 Domain annotation of proteins with GH domain targeting cellulose, xylan, and chitin identified in sequenced bacterial genomes.
10.1186/s13068-016-0538-6 Multi-Activity GH5.
10.1186/s13068-016-0538-6 Multi-Activity GH6.
10.1186/s13068-016-0538-6 Multi-Activity GH8.
10.1186/s13068-016-0538-6 Multi-Activity GH9.
10.1186/s13068-016-0538-6 Multi-Activity GH10.
10.1186/s13068-016-0538-6 Multi-Activity GH11.
10.1186/s13068-016-0538-6 Multi-Activity GH12. x10.1186/s13068-016-0538-6 Multi-Activity GH18.
10.1186/s13068-016-0538-6 Multi-Activity GH19.
10.1186/s13068-016-0538-6 Multi-Activity GH44.
10.1186/s13068-016-0538-6 Multi-Activity GH48.
10.1186/s13068-016-0538-6 GHs for cellulose, xylan, and chitin in *Opitutaceae bacterium* TAV1 (phylum Verrucomicrobia).
10.1186/s13068-016-0538-6 GHs for cellulose, xylan, and chitin in *Amycolatopsis mediterranei* U32 (phylum Actinobacteria).
10.1186/s13068-016-0538-6 GHs for cellulose, xylan, and chitin in *Micromonospora* (phylum Actinobacteria).
10.1186/s13068-016-0538-6 GHs for cellulose, xylan, and chitin in *Spirochaeta* (phylum Spirochaetes).
10.1186/s13068-016-0538-6 GHs for cellulose, xylan, and chitin in *Anaerophaga* (phylum Bacteroidetes).
10.1186/s13068-016-0538-6 GHs for cellulose, xylan, and chitin in *Asticcacaulis* (phylum Alpha-proteobacterium).
10.1186/s13068-016-0538-6 GHs for cellulose, xylan, and chitin in *Hamadaea tsunoensis* DSM44101 (phylum Actinobacteria).
10.1186/s13068-016-0538-6 GHs for cellulose, xylan, and chitin in *Cystobacter fuscus* DSM2262 (phylum Deltaproteobacteria).
10.1186/s13068-016-0538-6 GHs for cellulose, xylan, and chitin in *Catelliglobosispora koreensis* DSM44566 (phylum Actinobacteria).
10.1186/s13068-016-0538-6 GHs for cellulose, xylan, and chitin in *Sporocytophaga myxococcoides* DSM11118 (phylum Bacteroidetes).
10.1186/s13068-016-0538-6 GHs for cellulose, xylan, and chitin in *Kitasatospora setae* KM-6054 (phylum Actinobacteria).
10.1186/s13068-016-0538-6 GHs for cellulose, xylan, and chitin in *Niastalla koreensis* GH20-10 (phylum Bacteroidetes).
10.1186/s13068-016-0538-6 GHs for cellulose, xylan, and chitin in *Microbispora sp*. ATCC-PTA-5024 (phylum Actinobacteria).
10.1186/s13068-016-0538-6 Protein domains (i.e., PFam ID, target name, and PFam length), from PFam database, identified in this study. #hits: number of identified domains; and distribution of hit’s E-values.
10.1186/s13068-016-0538-6 Mantel correlation test (999 permutations) for the clustering of bacterial genomes according to GH-proteins and GH-domains distribution within bacterial genera with at least 3 sequenced genomes.
10.1186/s13068-016-0538-6 Clustering of sequenced 131 stains from the genus *Xanthomonas* (phylum Actinobacteria) according to the distribution of protein with GH domain for cellulose, xylan, and chitin (A) and the corresponding GH domains only (B).
10.1186/s13068-016-0538-6 Multi-activity GH with 3 catalytic domains targeting cellulose, xylan and chitin.

## Abbreviations

CAZy: carbohydrate-active enzyme
CBM: carbohydrate-binding domain
GH: glycosyl hydrolase
GT: glycosyl transferase
MA-GH: multi activity glycoside hydrolase
MD-GH: multi domain glycoside hydrolase
