# Supplementary material for: Natural diversity of cellulases, xylanases, and chitinases in bacteria
Source: Biotechnol Biofuels. 2016 Jun 29;9:133. doi: 10.1186/s13068-016-0538-6 (PMC4928363; doi:10.1186/s13068-016-0538-6)
Supplement: Supplementary file 26 — 10.1186/s13068-016-0538-6 Protein domains (i.e., PFam ID, target name, and PFam length), from PFam database, identified in this study. #hits: number of identified domains; and distribution of hit’s E-values. [file 13068_2016_538_MOESM26_ESM.docx]

Table S1. Protein domains (i.e., PFam ID, target name, and PFam length), from PFam database, identified in this study. #hits: number of identified domains; and distribution of hit’s E-values.

| **PFam ID** | **Target name** | **PFlength (AA)** | **#hits** | **Mean**  **E-value** | **Min**  **E-value** | **Max**  **E-value** |
| --- | --- | --- | --- | --- | --- | --- |
| PF10417 | 1-cysPrx_C | 40 | 243 | 7.37E-10 | 6.90E-14 | 5.00E-08 |
| PF05270 | AbfB | 140 | 5 | 1.08E-46 | 1.00E-46 | 1.10E-46 |
| PF02230 | Abhydrolase_2 | 217 | 2 | 7.20E-07 | 5.50E-07 | 8.90E-07 |
| PF07859 | Abhydrolase_3 | 211 | 5 | 3.48E-08 | 3.40E-16 | 1.40E-07 |
| PF12695 | Abhydrolase_5 | 145 | 21 | 1.25E-06 | 9.90E-13 | 8.90E-06 |
| PF00578 | AhpC-TSA | 124 | 242 | 2.64E-17 | 1.50E-34 | 6.40E-15 |
| PF00248 | Aldo_ket_red | 292 | 2 | 2.90E-62 | 2.90E-62 | 2.90E-62 |
| PF01261 | AP_endonuc_2 | 210 | 6 | 2.58E-06 | 3.10E-07 | 4.50E-06 |
| PF15780 | ASH | 98 | 28 | 7.14E-10 | 1.70E-54 | 2.00E-08 |
| PF12951 | Autotrns_rpt | 30 | 3 | 2.00E-10 | 2.00E-10 | 2.00E-10 |
| PF05448 | AXE1 | 317 | 2 | 4.20E-30 | 3.10E-30 | 5.30E-30 |
| PF01453 | B_lectin | 107 | 14 | 7.71E-17 | 3.50E-30 | 5.40E-16 |
| PF13004 | BACON | 53 | 93 | 1.91E-07 | 3.20E-23 | 6.90E-06 |
| PF13229 | Beta_helix | 158 | 5 | 1.84E-08 | 5.60E-19 | 4.60E-08 |
| PF13364 | BetaGal_dom4_5 | 110 | 6 | 4.33E-07 | 1.90E-09 | 1.30E-06 |
| PF02369 | Big_1 | 99 | 9 | 2.80E-13 | 1.20E-13 | 1.20E-12 |
| PF02368 | Big_2 | 79 | 54 | 2.75E-07 | 5.20E-43 | 6.70E-06 |
| PF07523 | Big_3 | 68 | 855 | 1.23E-07 | 2.20E-36 | 9.10E-06 |
| PF13750 | Big_3_3 | 158 | 2 | 2.40E-14 | 2.40E-14 | 2.40E-14 |
| PF13754 | Big_3_4 | 65 | 160 | 6.87E-07 | 7.00E-125 | 9.00E-06 |
| PF16640 | Big_3_5 | 90 | 58 | 8.63E-07 | 5.60E-42 | 4.40E-06 |
| PF07532 | Big_4 | 59 | 26 | 9.89E-08 | 1.50E-32 | 5.20E-07 |
| PF13205 | Big_5 | 108 | 3 | 1.83E-07 | 1.10E-09 | 5.00E-07 |
| PF06832 | BiPBP_C | 89 | 90 | 1.47E-06 | 1.50E-15 | 8.00E-06 |
| PF02012 | BNR | 12 | 57 | 1.42E-06 | 1.40E-12 | 4.50E-06 |
| PF14873 | BNR_assoc_N | 153 | 3 | 3.20E-23 | 4.60E-25 | 9.50E-23 |
| PF00028 | Cadherin | 93 | 5 | 2.20E-08 | 2.20E-18 | 1.10E-07 |
| PF16184 | Cadherin_3 | 144 | 4 | 4.47E-08 | 4.70E-10 | 8.90E-08 |
| PF12733 | Cadherin-like | 90 | 14 | 2.57E-08 | 3.10E-17 | 1.80E-07 |
| PF03160 | Calx-beta | 100 | 119 | 2.65E-09 | 1.90E-131 | 2.30E-07 |
| PF13715 | CarbopepD_reg_2 | 88 | 15 | 1.17E-06 | 1.60E-12 | 8.30E-06 |
| PF13620 | CarboxypepD_reg | 82 | 36 | 1.74E-07 | 6.10E-30 | 3.00E-06 |
| PF02013 | CBM_10 | 36 | 160 | 1.08E-06 | 2.00E-22 | 9.50E-06 |
| PF03425 | CBM_11 | 180 | 24 | 5.01E-11 | 1.20E-68 | 1.20E-09 |
| PF03426 | CBM_15 | 163 | 2 | 7.50E-87 | 7.60E-96 | 1.50E-86 |
| PF03424 | CBM_17_28 | 204 | 44 | 3.05E-43 | 2.90E-125 | 9.40E-42 |
| PF00553 | CBM_2 | 101 | 2448 | 7.47E-09 | 3.60E-64 | 8.70E-06 |
| PF00686 | CBM_20 | 97 | 2 | 6.40E-20 | 6.40E-20 | 6.40E-20 |
| PF00942 | CBM_3 | 82 | 779 | 1.41E-07 | 4.40E-74 | 9.40E-06 |
| PF16990 | CBM_35 | 121 | 134 | 4.41E-07 | 2.90E-44 | 9.30E-06 |
| PF02018 | CBM_4_9 | 131 | 1293 | 5.80E-08 | 1.80E-91 | 9.60E-06 |
| PF02839 | CBM_5_12 | 42 | 5069 | 8.57E-08 | 3.60E-36 | 9.90E-06 |
| PF14600 | CBM_5_12_2 | 62 | 898 | 8.51E-07 | 5.00E-42 | 7.70E-06 |
| PF03422 | CBM_6 | 125 | 385 | 7.97E-08 | 7.20E-102 | 7.70E-06 |
| PF03442 | CBM_X2 | 83 | 181 | 4.63E-09 | 4.80E-100 | 7.50E-07 |
| PF09212 | CBM27 | 170 | 20 | 6.50E-19 | 2.20E-100 | 1.30E-17 |
| PF09478 | CBM49 | 81 | 51 | 1.71E-06 | 3.60E-14 | 9.70E-06 |
| PF16841 | CBM60 | 93 | 103 | 4.49E-07 | 4.50E-67 | 8.10E-06 |
| PF06452 | CBM9_1 | 185 | 209 | 8.87E-18 | 2.50E-131 | 1.70E-15 |
| PF02927 | CelD_N | 86 | 1771 | 8.00E-08 | 1.70E-32 | 9.70E-06 |
| PF03174 | CHB_HEX_C | 75 | 57 | 7.02E-15 | 2.90E-124 | 2.00E-13 |
| PF13290 | CHB_HEX_C_1 | 67 | 54 | 5.38E-15 | 1.00E-113 | 2.90E-13 |
| PF06483 | ChiC | 174 | 942 | 2.72E-10 | 1.40E-83 | 5.10E-08 |
| PF03067 | Chitin_bind_3 | 169 | 7 | 3.43E-15 | 2.90E-35 | 2.20E-14 |
| PF08329 | ChitinaseA_N | 133 | 735 | 9.52E-15 | 3.80E-62 | 2.10E-12 |
| PF07538 | ChW | 35 | 3 | 1.90E-45 | 1.90E-45 | 1.90E-45 |
| PF08547 | CIA30 | 160 | 10 | 1.64E-06 | 2.50E-07 | 5.20E-06 |
| PF05738 | Cna_B | 70 | 5 | 2.06E-07 | 1.70E-07 | 3.50E-07 |
| PF00963 | Cohesin | 141 | 7 | 2.90E-238 | 2.90E-238 | 2.90E-238 |
| PF01391 | Collagen | 60 | 2 | 3.60E-07 | 3.60E-07 | 3.60E-07 |
| PF00030 | Crystall | 82 | 6 | 6.83E-09 | 4.40E-22 | 2.10E-08 |
| PF08964 | Crystall_3 | 86 | 3 | 2.10E-07 | 2.10E-07 | 2.10E-07 |
| PF07833 | Cu_amine_oxidN1 | 93 | 209 | 4.36E-07 | 4.70E-38 | 8.00E-06 |
| PF01473 | CW_binding_1 | 19 | 43 | 3.16E-07 | 1.10E-42 | 1.80E-06 |
| PF04122 | CW_binding_2 | 70 | 87 | 6.21E-50 | 2.10E-63 | 1.80E-48 |
| PF00404 | Dockerin_1 | 21 | 1102 | 1.51E-07 | 4.50E-19 | 8.90E-06 |
| PF03330 | DPBB_1 | 83 | 17 | 1.21E-06 | 2.10E-09 | 1.80E-06 |
| PF06439 | DUF1080 | 185 | 2 | 6.56E-08 | 1.10E-09 | 1.30E-07 |
| PF01345 | DUF11 | 76 | 3 | 1.82E-06 | 2.60E-07 | 2.60E-06 |
| PF07610 | DUF1573 | 44 | 23 | 3.76E-15 | 2.20E-40 | 9.60E-15 |
| PF08522 | DUF1735 | 73 | 29 | 5.25E-07 | 1.20E-18 | 6.50E-06 |
| PF03382 | DUF285 | 121 | 4 | 3.50E-26 | 3.50E-26 | 3.50E-26 |
| PF03629 | DUF303 | 230 | 6 | 4.73E-09 | 5.20E-78 | 1.10E-08 |
| PF13200 | DUF4015 | 315 | 2 | 6.00E-13 | 5.90E-13 | 6.10E-13 |
| PF13204 | DUF4038 | 309 | 4 | 1.73E-06 | 1.70E-23 | 2.90E-06 |
| PF13946 | DUF4214 | 72 | 36 | 1.47E-17 | 9.50E-53 | 1.40E-16 |
| PF14488 | DUF4434 | 167 | 9 | 3.69E-06 | 8.80E-07 | 8.60E-06 |
| PF16141 | DUF4849 | 321 | 70 | 9.31E-09 | 5.90E-115 | 6.50E-07 |
| PF16355 | DUF4982 | 61 | 32 | 4.18E-06 | 5.30E-08 | 9.40E-06 |
| PF16403 | DUF5011 | 106 | 247 | 1.21E-09 | 4.80E-48 | 1.70E-07 |
| PF16586 | DUF5060 | 80 | 11 | 1.28E-07 | 2.60E-15 | 1.20E-06 |
| PF00756 | Esterase | 251 | 16 | 6.95E-08 | 1.10E-29 | 5.70E-07 |
| PF10503 | Esterase_phd | 220 | 5 | 6.98E-19 | 4.90E-19 | 8.00E-19 |
| PF00754 | F5_F8_type_C | 127 | 428 | 4.02E-07 | 9.30E-124 | 9.70E-06 |
| PF06268 | Fascin | 111 | 6 | 1.48E-06 | 5.30E-14 | 8.50E-06 |
| PF01839 | FG-GAP | 37 | 2 | 3.50E-19 | 3.50E-19 | 3.50E-19 |
| PF00630 | Filamin | 101 | 3 | 1.91E-06 | 2.00E-08 | 5.10E-06 |
| PF09479 | Flg_new | 65 | 3 | 2.17E-08 | 1.50E-12 | 6.50E-08 |
| PF13860 | FlgD_ig | 81 | 8 | 1.04E-06 | 3.80E-11 | 5.40E-06 |
| PF00041 | fn3 | 85 | 2293 | 9.72E-08 | 2.30E-55 | 9.40E-06 |
| PF16893 | fn3_2 | 89 | 247 | 1.08E-06 | 2.40E-17 | 9.20E-06 |
| PF13287 | Fn3_assoc | 59 | 55 | 1.49E-12 | 2.50E-98 | 8.20E-11 |
| PF14310 | Fn3-like | 71 | 2 | 7.00E-11 | 9.80E-26 | 1.40E-10 |
| PF07501 | G5 | 75 | 387 | 8.37E-09 | 2.20E-34 | 1.30E-07 |
| PF16030 | GD_N | 108 | 3 | 2.47E-06 | 1.20E-06 | 3.10E-06 |
| PF03009 | GDPD | 259 | 2 | 1.95E-14 | 1.20E-32 | 3.90E-14 |
| PF13653 | GDPD_2 | 30 | 2 | 6.80E-08 | 6.80E-08 | 6.80E-08 |
| PF00331 | GH_10 | 316 | 2541 | 3.17E-09 | 2.40E-215 | 7.70E-06 |
| PF00457 | GH_11 | 178 | 534 | 6.24E-10 | 1.20E-132 | 3.00E-07 |
| PF03537 | GH_114 | 230 | 2 | 1.04E-82 | 9.70E-83 | 1.10E-82 |
| PF01670 | GH_12 | 214 | 2515 | 9.57E-11 | 2.90E-88 | 7.10E-08 |
| PF00722 | GH_16 | 177 | 8 | 1.30E-40 | 3.00E-44 | 8.30E-40 |
| PF00704 | GH_18 | 282 | 12771 | 3.28E-08 | 1.10E-229 | 9.90E-06 |
| PF00182 | GH_19 | 232 | 1683 | 2.14E-09 | 5.60E-86 | 3.60E-06 |
| PF02836 | GH_2_C | 302 | 17 | 5.84E-07 | 6.30E-14 | 7.30E-06 |
| PF02837 | GH_2_N | 171 | 2 | 3.90E-07 | 5.80E-30 | 7.80E-07 |
| PF02156 | GH_26 | 311 | 23 | 1.36E-38 | 7.80E-100 | 3.10E-37 |
| PF00933 | GH_3 | 319 | 15 | 2.67E-42 | 1.20E-84 | 4.00E-41 |
| PF01915 | GH_3_C | 186 | 15 | 3.76E-26 | 1.60E-51 | 1.70E-25 |
| PF02055 | GH_30 | 496 | 1361 | 9.80E-10 | 5.30E-115 | 8.90E-07 |
| PF01301 | GH_35 | 316 | 17 | 1.54E-07 | 5.40E-121 | 1.20E-06 |
| PF01229 | GH_39 | 486 | 7 | 5.43E-86 | 9.00E-169 | 3.80E-85 |
| PF02449 | GH_42 | 374 | 25 | 1.64E-07 | 8.10E-146 | 3.60E-06 |
| PF08533 | GH_42C | 58 | 6 | 4.57E-07 | 3.30E-10 | 1.30E-06 |
| PF08532 | GH_42M | 207 | 47 | 7.54E-08 | 1.00E-63 | 9.70E-07 |
| PF04616 | GH_43 | 288 | 48 | 1.00E-22 | 1.60E-83 | 4.80E-21 |
| PF12891 | GH_44 | 233 | 143 | 2.17E-13 | 9.80E-86 | 3.10E-11 |
| PF02015 | GH_45 | 210 | 31 | 2.77E-18 | 3.00E-48 | 4.30E-17 |
| PF02011 | GH_48 | 622 | 346 | 8.67E-152 | 0 | 3.00E-149 |
| PF00150 | GH_5 | 281 | 7908 | 2.66E-07 | 2.10E-127 | 9.90E-06 |
| PF07745 | GH_53 | 340 | 95 | 4.25E-60 | 5.70E-206 | 2.90E-58 |
| PF01341 | GH_6 | 304 | 3088 | 8.17E-26 | 3.60E-197 | 1.80E-22 |
| PF03664 | GH_62 | 272 | 32 | 3.15E-63 | 1.30E-166 | 3.30E-62 |
| PF01270 | GH_8 | 342 | 5003 | 1.84E-16 | 5.60E-154 | 2.80E-13 |
| PF03644 | GH_85 | 292 | 941 | 1.14E-13 | 2.60E-91 | 5.20E-11 |
| PF00759 | GH_9 | 410 | 2307 | 1.43E-10 | 9.80E-150 | 2.30E-07 |
| PF11790 | GH_cc | 238 | 5 | 2.44E-06 | 2.50E-07 | 5.60E-06 |
| PF02638 | GHL10 | 311 | 30 | 2.64E-27 | 1.60E-99 | 4.70E-26 |
| PF14871 | GHL6 | 137 | 5 | 1.66E-06 | 2.70E-07 | 5.00E-06 |
| PF14587 | Glyco_hydr_30_2 | 384 | 9 | 9.90E-07 | 6.10E-68 | 8.90E-06 |
| PF10111 | Glyco_tranf_2_2 | 281 | 50 | 9.98E-08 | 4.60E-15 | 2.30E-06 |
| PF13641 | Glyco_tranf_2_3 | 229 | 334 | 4.59E-24 | 4.30E-47 | 1.50E-21 |
| PF13692 | Glyco_trans_1_4 | 135 | 3 | 1.87E-13 | 3.40E-28 | 2.80E-13 |
| PF13632 | Glyco_trans_2_3 | 193 | 332 | 7.64E-18 | 1.40E-30 | 2.20E-15 |
| PF13579 | Glyco_trans_4_4 | 160 | 2 | 7.00E-12 | 1.30E-17 | 1.40E-11 |
| PF13506 | Glyco_transf_21 | 174 | 325 | 2.37E-12 | 3.00E-28 | 2.50E-10 |
| PF00534 | Glycos_transf_1 | 172 | 2 | 7.50E-12 | 4.90E-35 | 1.50E-11 |
| PF00535 | Glycos_transf_2 | 170 | 332 | 3.74E-22 | 5.00E-37 | 4.50E-20 |
| PF10648 | Gmad2 | 86 | 4 | 3.00E-07 | 3.00E-07 | 3.00E-07 |
| PF00746 | Gram_pos_anchor | 38 | 5 | 3.22E-06 | 1.30E-06 | 7.80E-06 |
| PF02518 | HATPase_c | 106 | 2 | 1.01E-20 | 3.10E-21 | 1.70E-20 |
| PF14501 | HATPase_c_5 | 101 | 3 | 2.27E-07 | 2.00E-07 | 2.40E-07 |
| PF05345 | He_PIG | 49 | 5 | 1.60E-09 | 6.00E-17 | 8.00E-09 |
| PF02985 | HEAT | 31 | 7 | 2.69E-10 | 5.10E-26 | 9.40E-10 |
| PF13646 | HEAT_2 | 88 | 4 | 2.75E-13 | 8.80E-40 | 1.10E-12 |
| PF13513 | HEAT_EZ | 55 | 5 | 6.30E-06 | 6.30E-06 | 6.30E-06 |
| PF03130 | HEAT_PBS | 27 | 5 | 2.70E-12 | 2.70E-12 | 2.70E-12 |
| PF00353 | HemolysinCabind | 18 | 72 | 4.63E-07 | 2.90E-32 | 4.20E-06 |
| PF00512 | HisKA | 64 | 2 | 2.30E-13 | 1.80E-13 | 2.80E-13 |
| PF02494 | HYR | 81 | 5 | 1.11E-08 | 4.60E-11 | 2.70E-08 |
| PF07679 | I-set | 90 | 12 | 8.96E-08 | 1.50E-25 | 5.30E-07 |
| PF00047 | ig | 87 | 2 | 1.70E-09 | 1.70E-09 | 1.70E-09 |
| PF13895 | Ig_2 | 80 | 11 | 9.58E-07 | 1.30E-12 | 9.30E-06 |
| PF13927 | Ig_3 | 69 | 10 | 1.85E-06 | 1.50E-10 | 8.60E-06 |
| PF02210 | Laminin_G_2 | 127 | 6 | 2.02E-06 | 4.00E-19 | 6.60E-06 |
| PF13385 | Laminin_G_3 | 152 | 103 | 1.48E-07 | 7.60E-64 | 3.00E-06 |
| PF00057 | Ldl_recept_a | 37 | 2 | 1.40E-18 | 1.40E-18 | 1.40E-18 |
| PF08310 | LGFP | 53 | 6 | 2.74E-06 | 7.20E-11 | 4.90E-06 |
| PF00657 | Lipase_GDSL | 163 | 23 | 7.12E-07 | 2.30E-18 | 9.40E-06 |
| PF13472 | Lipase_GDSL_2 | 188 | 30 | 3.24E-09 | 7.00E-15 | 2.70E-08 |
| PF12799 | LRR_4 | 43 | 27 | 6.64E-20 | 1.80E-37 | 4.60E-19 |
| PF13306 | LRR_5 | 128 | 4 | 3.00E-07 | 5.80E-17 | 1.20E-06 |
| PF13855 | LRR_8 | 61 | 13 | 6.17E-12 | 3.30E-26 | 3.70E-11 |
| PF08309 | LVIVD | 42 | 9 | 2.10E-26 | 2.10E-26 | 2.10E-26 |
| PF01476 | LysM | 44 | 1931 | 1.42E-08 | 3.60E-117 | 3.30E-06 |
| PF04397 | LytTR | 98 | 8 | 1.49E-08 | 7.10E-13 | 1.10E-07 |
| PF11721 | Malectin | 174 | 17 | 2.65E-25 | 5.20E-34 | 2.50E-24 |
| PF13425 | O-antigen_lig | 140 | 3 | 5.39E-12 | 5.80E-13 | 1.50E-11 |
| PF07691 | PA14 | 146 | 10 | 2.62E-15 | 2.80E-42 | 2.50E-14 |
| PF00934 | PE | 93 | 9 | 3.29E-16 | 2.50E-36 | 1.90E-15 |
| PF00326 | Peptidase_S9 | 212 | 11 | 2.87E-07 | 1.60E-09 | 2.90E-06 |
| PF01471 | PG_binding_1 | 57 | 35 | 4.64E-07 | 2.40E-32 | 7.80E-06 |
| PF16655 | PhoD_N | 90 | 5 | 1.72E-06 | 4.60E-07 | 3.60E-06 |
| PF07238 | PilZ | 103 | 4 | 1.46E-13 | 3.40E-15 | 3.10E-13 |
| PF00801 | PKD | 71 | 428 | 1.01E-06 | 2.30E-59 | 9.70E-06 |
| PF16820 | PKD_3 | 68 | 70 | 2.74E-06 | 6.40E-08 | 9.50E-06 |
| PF00069 | Pkinase | 262 | 2 | 2.85E-38 | 2.70E-41 | 5.70E-38 |
| PF07714 | Pkinase_Tyr | 260 | 2 | 8.62E-21 | 2.40E-22 | 1.70E-20 |
| PF01357 | Pollen_allerg_1 | 80 | 2 | 1.98E-07 | 5.60E-08 | 3.40E-07 |
| PF01522 | Polysacc_deac_1 | 124 | 454 | 5.55E-08 | 1.10E-34 | 9.10E-06 |
| PF04886 | PT | 36 | 8 | 2.40E-06 | 5.70E-10 | 5.20E-06 |
| PF16656 | Pur_ac_phosph_N | 94 | 89 | 1.61E-06 | 1.80E-18 | 9.10E-06 |
| PF08534 | Redoxin | 148 | 241 | 3.32E-11 | 2.40E-19 | 8.00E-09 |
| PF00581 | Rhodanese | 107 | 2 | 1.11E-13 | 2.20E-15 | 2.20E-13 |
| PF00652 | Ricin_B_lectin | 126 | 538 | 2.19E-09 | 4.10E-47 | 6.90E-07 |
| PF14200 | RicinB_lectin_2 | 105 | 722 | 1.62E-10 | 2.20E-51 | 8.60E-08 |
| PF12791 | RsgI_N | 53 | 7 | 1.25E-11 | 2.30E-12 | 7.40E-11 |
| PF07738 | Sad1_UNC | 135 | 2 | 1.85E-06 | 1.80E-06 | 1.90E-06 |
| PF08239 | SH3_3 | 55 | 278 | 6.66E-07 | 1.20E-22 | 9.00E-06 |
| PF06347 | SH3_4 | 56 | 28 | 2.25E-06 | 3.30E-08 | 9.60E-06 |
| PF00395 | SLH | 45 | 727 | 5.78E-13 | 1.90E-47 | 1.80E-10 |
| PF16967 | TcfC | 68 | 3 | 1.50E-09 | 1.50E-09 | 1.50E-09 |
| PF06283 | ThuA | 210 | 15 | 9.23E-45 | 1.30E-47 | 1.30E-43 |
| PF01833 | TIG | 85 | 3 | 9.13E-09 | 1.40E-09 | 1.30E-08 |
| PF01108 | Tissue_fac | 107 | 20 | 1.89E-06 | 3.00E-11 | 4.60E-06 |
| PF12371 | TMEM131_like | 84 | 20 | 6.95E-08 | 7.80E-09 | 1.20E-07 |
| PF00515 | TPR_1 | 34 | 3 | 5.80E-08 | 5.80E-08 | 5.80E-08 |
| PF13414 | TPR_11 | 68 | 4 | 8.30E-15 | 8.30E-15 | 8.30E-15 |
| PF13424 | TPR_12 | 79 | 7 | 2.60E-09 | 2.60E-09 | 2.60E-09 |
| PF13428 | TPR_14 | 44 | 7 | 1.20E-07 | 1.20E-07 | 1.20E-07 |
| PF13432 | TPR_16 | 65 | 5 | 1.30E-24 | 1.30E-24 | 1.30E-24 |
| PF14559 | TPR_19 | 68 | 8 | 1.40E-22 | 1.40E-22 | 1.40E-22 |
| PF07719 | TPR_2 | 34 | 8 | 6.00E-12 | 6.00E-12 | 6.00E-12 |
| PF02412 | TSP_3 | 36 | 2 | 2.00E-08 | 2.00E-08 | 2.00E-08 |
| PF13517 | VCBS | 61 | 11 | 4.38E-07 | 6.30E-31 | 2.40E-06 |
| PF07483 | W_rich_C | 105 | 5 | 5.10E-99 | 5.10E-99 | 5.10E-99 |
| PF04932 | Wzy_C | 156 | 3 | 1.92E-14 | 1.50E-15 | 2.80E-14 |
| PF07495 | Y_Y_Y | 66 | 48 | 2.78E-06 | 1.70E-24 | 7.50E-06 |
| PF07949 | YbbR | 82 | 4 | 1.10E-06 | 1.10E-06 | 1.10E-06 |
| PF04650 | YSIRK_signal | 26 | 171 | 1.54E-10 | 8.90E-13 | 1.70E-09 |
