# Supplementary material for: Natural diversity of cellulases, xylanases, and chitinases in bacteria
Source: Biotechnol Biofuels. 2016 Jun 29;9:133. doi: 10.1186/s13068-016-0538-6 (PMC4928363; doi:10.1186/s13068-016-0538-6)
Supplement: Supplementary file 27 — 10.1186/s13068-016-0538-6 Mantel correlation test (999 permutations) for the clustering of bacterial genomes according to GH-proteins and GH-domains distribution within bacterial genera with at least 3 sequenced genomes. [file 13068_2016_538_MOESM27_ESM.docx]

Table S2. Mantel correlation test (999 permutations) for the clustering of bacterial genomes according to GH-proteins and GH-domains distribution within bacterial genera with at least 3 sequenced genomes.

| Genus | R.mantel | P.mantel | N genomes | Phylum |
| --- | --- | --- | --- | --- |
| *Acidaminococcus* | 1.000 | 0.167 | 6 | Firmicutes |
| *Acidithiobacillus* | 1.000 | 0.017 | 5 | Gammaproteobacteria |
| *Acidovorax* | 0.872 | 0.002 | 8 | Betaproteobacteria |
| *Actinomyces* | 1.000 | 0.006 | 6 | Actinobacteria |
| *Aeromonas* | 0.800 | 0.001 | 31 | Gammaproteobacteria |
| *Agrobacterium* | 1.000 | 0.001 | 12 | Alphaproteobacteria |
| *Algoriphagus* | 1.000 | 0.001 | 6 | Bacteroidetes.Chlorobi_group |
| *Alicyclobacillus* | 0.850 | 0.001 | 9 | Firmicutes |
| *Aliivibrio* | 0.119 | 0.583 | 4 | Gammaproteobacteria |
| *Alistipes* | 0.847 | 0.001 | 7 | Bacteroidetes.Chlorobi_group |
| *Alteromonas* | 1.000 | 0.001 | 15 | Gammaproteobacteria |
| *Amycolatopsis* | 0.930 | 0.001 | 17 | Actinobacteria |
| *Anabaena* | 1.000 | 0.167 | 4 | Cyanobacteria |
| *Anaeromyxobacter* | 1.000 | 0.167 | 4 | delta_epsilon_subdivisions |
| *Arthrobacter* | 0.848 | 0.001 | 17 | Actinobacteria |
| *Asticcacaulis* | 0.992 | 0.002 | 7 | Alphaproteobacteria |
| *Azospirillum* | 0.981 | 0.001 | 9 | Alphaproteobacteria |
| *Bacillus* | 0.827 | 0.001 | 412 | Firmicutes |
| *Bacteroides* | 0.817 | 0.001 | 88 | Bacteroidetes.Chlorobi_group |
| *Bifidobacterium* | 0.720 | 0.001 | 57 | Actinobacteria |
| *Blautia* | 0.290 | 0.067 | 6 | Firmicutes |
| *Bordetella* | 1.000 | 0.250 | 4 | Betaproteobacteria |
| *Bradyrhizobium* | 0.988 | 0.001 | 57 | Alphaproteobacteria |
| *Brevibacillus* | 0.416 | 0.004 | 13 | Firmicutes |
| *Brevundimonas* | 1.000 | 0.012 | 7 | Alphaproteobacteria |
| *Burkholderia* | 0.934 | 0.001 | 61 | Betaproteobacteria |
| *Butyrivibrio* | 0.785 | 0.001 | 30 | Firmicutes |
| *Caldicellulosiruptor* | 0.554 | 0.002 | 10 | Firmicutes |
| *Capnocytophaga* | 0.950 | 0.001 | 18 | Bacteroidetes.Chlorobi_group |
| *Caulobacter* | 0.928 | 0.001 | 9 | Alphaproteobacteria |
| *Cellulomonas* | 0.430 | 0.117 | 5 | Actinobacteria |
| *Cellulophaga* | 0.636 | 0.001 | 7 | Bacteroidetes.Chlorobi_group |
| *Chlorobium* | 1.000 | 0.083 | 4 | Bacteroidetes.Chlorobi_group |
| *Chloroflexus* | 0.844 | 0.250 | 4 | Chloroflexi |
| *Chryseobacterium* | 0.938 | 0.002 | 7 | Bacteroidetes.Chlorobi_group |
| *Citrobacter* | 0.946 | 0.002 | 16 | Gammaproteobacteria |
| *Clostridium* | 0.921 | 0.001 | 411 | Firmicutes |
| *Coprobacillus* | 0.526 | 0.033 | 5 | Firmicutes |
| *Coprococcus* | 0.637 | 0.167 | 4 | Firmicutes |
| *Corynebacterium* | 1.000 | 0.002 | 18 | Actinobacteria |
| *Coxiella* | 1.000 | 0.013 | 9 | Gammaproteobacteria |
| *Cronobacter* | 0.833 | 0.001 | 30 | Gammaproteobacteria |
| *Cupriavidus* | 1.000 | 0.001 | 12 | Betaproteobacteria |
| *Cyanothece* | 1.000 | 0.033 | 6 | Cyanobacteria |
| *Cytophaga* | 0.920 | 0.167 | 4 | Bacteroidetes.Chlorobi_group |
| *Deinococcus* | 0.506 | 0.023 | 7 | Deinococcus-Thermus |
| *Desulfitobacterium* | 0.911 | 0.001 | 9 | Firmicutes |
| *Desulfotomaculum* | 0.498 | 0.027 | 10 | Firmicutes |
| *Desulfovibrio* | 0.609 | 0.014 | 9 | delta_epsilon_subdivisions |
| *Dickeya* | 0.961 | 0.001 | 32 | Gammaproteobacteria |
| *Dorea* | 0.217 | 0.167 | 6 | Firmicutes |
| *Dyadobacter* | 0.810 | 0.042 | 4 | Bacteroidetes.Chlorobi_group |
| *Enterobacter* | 0.741 | 0.001 | 94 | Gammaproteobacteria |
| *Enterococcus* | 0.731 | 0.001 | 373 | Firmicutes |
| *Erwinia* | 0.982 | 0.001 | 17 | Gammaproteobacteria |
| *Escherichia* | 0.999 | 0.001 | 1613 | Gammaproteobacteria |
| *Eubacterium* | 0.485 | 0.001 | 12 | Firmicutes |
| *Ferrimonas* | 0.638 | 0.167 | 4 | Gammaproteobacteria |
| *Flavobacterium* | 0.930 | 0.001 | 20 | Bacteroidetes.Chlorobi_group |
| *Francisella* | 0.374 | 0.001 | 42 | Gammaproteobacteria |
| *Frankia* | 1.000 | 0.001 | 9 | Actinobacteria |
| *Geobacillus* | 0.898 | 0.001 | 20 | Firmicutes |
| *Glaciecola* | 0.974 | 0.001 | 8 | Gammaproteobacteria |
| *Gluconacetobacter* | 1.000 | 0.002 | 10 | Alphaproteobacteria |
| *Gordonia* | 0.857 | 0.001 | 20 | Actinobacteria |
| *Halomonas* | 1.000 | 0.200 | 5 | Gammaproteobacteria |
| *Herbaspirillum* | 1.000 | 0.002 | 11 | Betaproteobacteria |
| *Klebsiella* | 0.982 | 0.001 | 260 | Gammaproteobacteria |
| *Labrenzia* | 1.000 | 0.250 | 4 | Alphaproteobacteria |
| *Lactobacillus* | 0.984 | 0.001 | 126 | Firmicutes |
| *Lactococcus* | 0.611 | 0.001 | 35 | Firmicutes |
| *Legionella* | 1.000 | 0.001 | 20 | Gammaproteobacteria |
| *Leifsonia* | 0.930 | 0.167 | 4 | Actinobacteria |
| *Leptospira* | 1.000 | 0.001 | 313 | Spirochaetes |
| *Leuconostoc* | 0.974 | 0.017 | 5 | Firmicutes |
| *Listeria* | 0.843 | 0.001 | 77 | Firmicutes |
| *Lysinibacillus* | 0.720 | 0.006 | 6 | Firmicutes |
| *Meiothermus* | 1.000 | 0.167 | 6 | Deinococcus-Thermus |
| *Mesorhizobium* | 1.000 | 0.001 | 71 | Alphaproteobacteria |
| *Methylobacter* | 1.000 | 0.167 | 4 | Gammaproteobacteria |
| *Methylobacterium* | 0.998 | 0.001 | 20 | Alphaproteobacteria |
| *Microbacterium* | 0.860 | 0.001 | 15 | Actinobacterium |
| *Micromonospora* | 0.865 | 0.017 | 5 | Actinobacteria |
| *Mycobacterium* | 0.993 | 0.001 | 2147 | Actinobacteria |
| *Myxococcus* | 0.943 | 0.050 | 5 | delta_epsilon_subdivisions |
| *Nocardia* | 1.000 | 0.001 | 15 | Actinobacteria |
| *Nocardioides* | 0.406 | 0.150 | 5 | Actinobacteria |
| *Nocardiopsis* | 0.725 | 0.001 | 20 | Actinobacteria |
| *Novosphingobium* | 0.864 | 0.002 | 8 | Alphaproteobacteria |
| *Oribacterium* | 1.000 | 0.005 | 8 | Firmicutes |
| *Paenibacillus* | 0.847 | 0.001 | 79 | Firmicutes |
| *Pantoea* | 1.000 | 0.001 | 23 | Gammaproteobacteria |
| *Parabacteroides* | 1.000 | 0.001 | 13 | Bacteroidetes.Chlorobi_group |
| *Pectobacterium* | 0.957 | 0.001 | 9 | Gammaproteobacteria |
| *Pediococcus* | 1.000 | 0.250 | 4 | Firmicutes |
| *Pedobacter* | 0.859 | 0.001 | 8 | Bacteroidetes.Chlorobi_group |
| *Photobacterium* | 0.625 | 0.001 | 10 | Gammaproteobacteria |
| *Photorhabdus* | 1.000 | 0.017 | 5 | Gammaproteobacteria |
| *Prevotella* | 0.730 | 0.001 | 58 | Bacteroidetes.Chlorobi_group |
| *Propionibacterium* | 1.000 | 0.001 | 110 | Actinobacteria |
| *Providencia* | 0.134 | 0.167 | 5 | Gammaproteobacteria |
| *Pseudoalteromonas* | 0.835 | 0.001 | 40 | Gammaproteobacteria |
| *Pseudomonas* | 0.851 | 0.001 | 480 | Gammaproteobacteria |
| *Pseudoxanthomonas* | 0.963 | 0.002 | 8 | Gammaproteobacteria |
| *Psychromonas* | 0.910 | 0.001 | 6 | Gammaproteobacteria |
| *Ralstonia* | 0.871 | 0.001 | 14 | Betaproteobacteria |
| *Rhizobium* | 1.000 | 0.001 | 69 | Alphaproteobacteria |
| *Rhodobacter* | 1.000 | 0.200 | 5 | Alphaproteobacteria |
| *Rhodococcus* | 0.959 | 0.001 | 21 | Actinobacteria |
| *Rhodopirellula* | 0.789 | 0.001 | 9 | Planctomycetes |
| *Rhodopseudomonas* | 1.000 | 0.121 | 8 | Alphaproteobacteria |
| *Roseburia* | 0.864 | 0.167 | 4 | Firmicutes |
| *Ruminococcus* | 0.788 | 0.001 | 23 | Firmicutes |
| *Saccharomonospora* | 1.000 | 0.010 | 8 | Actinobacteria |
| *Saccharopolyspora* | 0.999 | 0.050 | 5 | Actinobacteria |
| *Salinispora* | 0.810 | 0.001 | 94 | Actinobacteria |
| *Salmonella* | 0.939 | 0.001 | 617 | Gammaproteobacteria |
| *Selenomonas* | 1.000 | 0.067 | 6 | Firmicutes |
| *Serratia* | 0.981 | 0.001 | 37 | Gammaproteobacteria |
| *Shewanella* | 0.394 | 0.002 | 27 | Gammaproteobacteria |
| *Shigella* | 1.000 | 0.001 | 102 | Gammaproteobacteria |
| *Sinorhizobium* | 1.000 | 0.079 | 15 | Alphaproteobacteria |
| *Sphingobacterium* | 0.788 | 0.042 | 4 | Bacteroidetes.Chlorobi_group |
| *Sphingobium* | 0.972 | 0.001 | 17 | Alphaproteobacteria |
| *Sphingomonas* | 0.916 | 0.001 | 25 | Alphaproteobacteria |
| *Spirochaeta* | 0.996 | 0.002 | 7 | Spirochaetes |
| *Spirosoma* | 0.731 | 0.042 | 4 | Bacteroidetes.Chlorobi_group |
| *Staphylococcus* | 0.539 | 0.101 | 7 | Firmicutes |
| *Stenotrophomonas* | 1.000 | 0.001 | 18 | Gammaproteobacteria |
| *Streptococcus* | 0.558 | 0.001 | 648 | Firmicutes |
| *Streptomyces* | 0.709 | 0.001 | 135 | Actinobacteria |
| *Synechococcus* | 0.839 | 0.001 | 14 | Cyanobacteria |
| *Teredinibacter* | 0.626 | 0.005 | 8 | Gammaproteobacteria |
| *Thermoanaerobacter* | 0.885 | 0.001 | 16 | Firmicutes |
| *Thermoanaerobacterium* | 0.867 | 0.008 | 5 | Firmicutes |
| *Thermotoga* | 0.859 | 0.001 | 11 | Thermotogae |
| *Treponema* | 0.941 | 0.001 | 22 | Spirochaetes |
| *Vibrio* | 0.594 | 0.001 | 527 | Gammaproteobacteria |
| *Xanthomonas* | 0.957 | 0.001 | 131 | Gammaproteobacteria |
| *Xenorhabdus* | 1.000 | 0.100 | 5 | Gammaproteobacteria |
| *Xylella* | 0.814 | 0.001 | 14 | Gammaproteobacteria |
| *Yersinia* | 0.996 | 0.001 | 138 | Gammaproteobacteria |
